# Attributes of carbapenemase encoding conjugative plasmid pNDM-SAL from an extensively drug-resistant *Salmonella enterica* Serovar Senftenberg

**DOI:** 10.3389/fmicb.2015.00969

**Published:** 2015-09-15

**Authors:** Anirban Sarkar, Gururaja P. Pazhani, Goutam Chowdhury, Amit Ghosh, Thandavarayan Ramamurthy

**Affiliations:** ^1^Department of Bacteriology, National Institute of Cholera and Enteric DiseasesKolkata, India; ^2^Centre for Drug Discovery and Development, Sathyabama UniversityChennai, India; ^3^Center for Human Microbial Ecology, Translational Health Science and Technology InstituteFaridabad, India

**Keywords:** NDM, carbapenemase, *S.* Senftenberg, enteric pathogens, pNDM-SAL

## Abstract

A carbapenem resistant *Salmonella enterica* serovar Senftenberg isolate BCH 2406 was isolated from a diarrheal child attending an outpatient unit of B.C. Roy Hospital in Kolkata, India. This isolate was positive for the *bla*_NDM-1_ in the PCR assay, which was confirmed by amplicon sequencing. Except for tetracycline, this isolate was resistant to all the tested antimicrobials. The *bla*_NDM-1_ was found to be located on a 146.13-kb mega plasmid pNDM-SAL, which could be conjugally transferred into *Escherichia coli* and other enteric pathogens such as *Vibrio cholerae* O1 Ogawa and *Shigella flexneri* 2a. However, the expression of β-lactam resistance is not the same in different bacteria. The whole genome sequence of pNDM-SAL was determined and compared with other pNDM plasmids available in public domain. This plasmid is an IncA/C incompatibility type composed of 155 predicted coding sequences and shares homology with plasmids of *E. coli* pNDM-1_Dok01, *Klebsiella* pNDM-KN, and *Citrobacter* pNDM-CIT. In pNDM-SAL, gene cluster containing *bla*_NDM-1_ was located between IS*26* and IS*4321* elements. Between the IS*26* element and the *bla*_NDM-1_, a truncated IS*Aba125* insertion sequence was identified. Downstream of the *bla*_NDM-1_, other genes, such as *ble*_MBL_, *trpF*, *tat*, and an IS*CR1* element with class 1 integron containing *aac(6′)-Ib* were detected. Another β-lactacamase gene, *bla*_CMY -4_ was found to be inserted in IS*1* element within the type IV conjugative transfer loci of the plasmid. This gene cluster had *blc* and *sugE* downstream of the *bla*_CMY -4_. From our findings, it appears that the strain *S.* Senftenberg could have acquired the NDM plasmid from the other members of Enterobacteriaceae. Transfer of NDM plasmids poses a danger in the management of infectious diseases.

## Introduction

Emergence of carbapenem resistance among Gram-negative bacteria is a major public health problem as they are associated with critical infections. This drug has been considered as one of the vital drugs against pathogens which produce extended spectrum β-lactamases (ESBLs). New Delhi Metallo-β-lactamase-1(NDM-1), which is a recent addition to the carbapenemase has become a major concern worldwide due to its rapid spread across different members of Enterobacteriaceae and other Gram-negative bacteria. The NDM-1 encoding gene (*bla*_NDM-1_) was first detected in *Klebsiella pneumoniae* and *Escherichia coli* recovered from a Swedish patient who had undergone treatment in New Delhi, India (Yong et al., 2009; Ghosh et al., 2014). Thereafter, this gene was identified in different Gram-negative bacteria in several countries including the USA, Canada, France, Sweden, UK, Germany, Japan, Austria, Africa, and Australia (Rolain et al., 2010). Presence of *bla*_NDM-1_ was generally associated with resistance to most of the antimicrobials, including fluoroquinolones, aminoglycosides, and β-lactams. The *bla*_NDM_ was found to be located of different large plasmids, which were readily transferable to other bacterial species (Kumarasamy et al., 2010; Rolain et al., 2010). The spread of the *bla*_NDM-1_ gene acquired by IncA/C MDR plasmids has drastically reduced the therapeutic options available to the physicians (Ghosh et al., 2014). In this study, we report the isolation of a *Salmonella enterica* serovar Senftenberg (*S.* Senftenberg) isolate carrying the *bla*_NDM-1_ gene on a large plasmid, which also possessed several genes responsible for extensively drug resistance (EDR).

## Materials and Methods

### Bacterial Strains

A carbapenem resistant *Salmonella enterica* isolate (BCH 2406) was isolated in 2012 from a five years old child who attended the outpatient department of B.C. Roy Memorial Hospital for Children, Kolkata for the treatment of diarrhea. This isolate was serotyped according to White-Kauffmann-Le Minor scheme with commercially available antisera (S&A Reagents Lab Ltd., Bangkok, Thailand). Tetracycline resistant XL1-Blue (TET^R^) and sodium azide resistant *E. coli* J53 (Az^R^) strains were used for conjugation experiments. In addition, ampicillin sensitive *Vibrio cholerae* O1 Ogawa (IDH 5313) and *Shigella flexneri* 2a (IDH 3077), isolated and identified from diarrheal patients were used as recipients. All the strains were preserved in Luria Bertani (LB) broth (Difco, Sparks, MD, USA) containing 15% glycerol at –80°C. Transconjugants were also maintained in nutrient agar (Difco) stab supplemented with 15 μg/ml ceftriaxone. *E. coli* ATCC 25922 was served as control in antimicrobial susceptibility testing.

### Detection of Carbapenem Resistance Encoding Gene

Presence of *bla*_NDM_ was identified by PCR with previously described primers (Chen et al., 2011) using Taq DNA polymerase (Roche, Mannheim, Germany). Amplicons were purified using a PCR product purification kit (Qiagen, Hilden, Germany) and sequenced using the ABI Big Dye terminator cycle sequencing ready reaction kit, version 3.1 (Applied Biosystems, Foster City, CA, USA) in an automated DNA sequencer (ABI 3730, Applied Biosystems). The sequences were assembled and analyzed using DNASTAR software (DNASTAR, Inc., Madison, WI, USA).

### Conjugation

To test the mobility and promiscuity of the *bla*_NDM-1_ haboring plasmid, conjugation by broth mating technique was carried out using NDM-positive *Salmonella* isolate as donor with four different recipients, namely *E. coli* XL1-Blue (TET^R^), *E. coli* J53 (Az^R^), *V. cholerae* O1 Ogawa, and *S. flexneri* 2a. In brief, overnight cultures of the bacteria were diluted in LB broth and allowed to grow as late-exponential phase culture. Cell density was adjusted to 1.5 × 10^8^cells/ml. Donor and recipient cells were mixed at 1:2 donor-to-recipient ratios in 1 ml of LB broth and allowed grow overnight at 37°C. In all cases, the donor and recipient suspensions were also diluted in phosphate buffer saline (PBS) with a dilution of 10^-3^ and 10^-5^ and plated on MacConkey agar (Difco) to confirm the purity and count the colonies.

To recover *bla*_NDM-1_ positive transconjugants, several selective media were used. Transconjugants (conjugally transferred, CT) of XL1-Blue (CT-*E. coli* XL1-Blue) and *S. flexneri* 2a (CT-*S. flexneri*) were selected on xylose lysine desoxycholate (XLD, Difco) agar supplemented with tetracycline (30 μg/ml) and ceftriaxone (5 μg/ml). Similarly, transconjugants of *V. cholerae* (CT-*V. cholerae*) were obtained with selection based on growth on ceftriaxone (5 μg/ml) containing thiosulphate citrate bile salts sucrose (TCBS, Eiken, Tokyo, Japan) agar and for transconjugants of *E. coli* J53 (CT-*E. coli* J53), MacConkey agar containing both ceftriaxone (5 μg/ml) and sodium azide (100 μg/ml) was used. Transconjugants were confirmed as *bla*_NDM-1_ positive by PCR analysis followed by PCR amplicon sequencing. The transfer frequencies were expressed as the number of transconjugants per donor cell.

### Antimicrobial Susceptibility Testing

To confirm the transfer of resistance phenotype, antibiotic susceptibility patterns of the donor, recipients, and transconjugants were determined after their growth on Mueller–Hinton (MH, Difco) agar by disk diffusion method in accordance with Clinical and Laboratory Standards Institute [CLSI] (2014) using commercially available disks (Becton Dickinson Company, Sparks, MD) namely, ampicillin (AMP), cefuroxime (CXM), ceftriaxone (CRO), cefotaxime (CTX), cefotaxime/clavulanic acid (CTX-CLA), ceftazidime (CAZ), ceftazidime/clavulanic acid (CAZ-CLA), chloramphenicol (CHL), nalidixic acid (NA), ciprofloxacin (CIP), ofloxacin (OFX), norfloxacin (NOR), imipenem (IPM), streptomycin (STR), azithromycin (AZM) tetracycline (TET), trimethoprim/sulfamethoxazole (SXT). To measure the increase of resistance in the transconjugants, MICs of antibiotics (ceftriaxone, imipenem, tetracycline, and sulfamethoxazole/trimethoprim) were determined by *E* test (AB bioMérieux, Solna, Sweden) in comparison with the wild NDM-positive *Salmonella* isolate.

### Plasmid analysis

Plasmid DNA was extracted from donor, recipients, and transconjugants by Kado and Liu’s (1981) method and analyzed by gel electrophoresis using 0.8% agarose. Presence of *bla*_NDM-1_ in the purified plasmid of the donor and transconjugants was determined by PCR. For size determination, plasmid analysis by S1 nuclease-pulsed-field gel electrophoresis (PFGE) (Barton et al., 1995) was performed with the donor and CT-*E. coli* J53. Total bacterial DNA prepared in agarose plugs was digested with S1 nuclease (Fermentas, Waltham, MA, USA) and separated using a CHEF-Mapper PFGE system (Bio-Rad, Hercules CA, USA), as reported previously (Kumarasamy et al., 2010). The PFGE conditions were run time 18 h gradient 6 V/cm, temperature 14°C, included angle 120° and initial and final pulses conducted for 2.16 s and 54.17 s, respectively. DNA of *S. enterica* serovar Braenderup H9812 digested with *Xba*1 (Roche) was included as control size marker. Incompatibility group of plasmid was determined for the *Salmonella* isolate and *E. coli* J53 transconjugant (as *E. coli* J53 Az^R^ was devoid of plasmids) using the PCR-based replicon typing (PBRT) method as described by Carattoli et al. (2005).

### Sequencing

Plasmid DNA was prepared from the *E. coli* J53 tranconjugant using Qiagen Maxiprep kit (Qiagen). Paired-end libraries (300–500 bp fragments) were constructed by using the Illumina *H* TruSeqTM DNA Sample preparation kit (Illumina). Each library was deposited onto a HiSeq Flow Cell and sequenced using an Illumina HiSeq-2000 next-generation DNA sequencer. The distributions of “base quality” “base composition” and %GC of the plasmid were checked. Based on these distributions, the first 15 bases and last one base were trimmed to avoid specific sequence bias and poor quality bases. Errors were corrected in the sequence data using the HiTEC tool. Contig assembly and predicted gaps were then confirmed and filled by PCR-based gap closure, confirmed by DNA sequencing of the amplicons (Applied Biosystems). To assemble the contigs, ABySS and Edena software were used by varying the parameter ‘k’ and ‘overlap cut-off’, respectively. This step produced several contigs for each parameter setting. The contigs were then integrated using contig integrator for sequence assembly (CISA). A blastN search was made against the ‘nt’ database for each contig and two contigs were retained for further downstream analysis. The open reading frames (ORFs) from the contigs were generated by CISA using Glimmer-MG program. For all these ORFs, the nucleotide sequence and amino acid sequences were obtained and translated in the appropriate frame. The predicted ORFs were annotated using an in-house pipeline (CANoPI-Contig Annotator Pipeline) that also includes blastX search for each ORF sequence against ‘nr’ database provided by NCBI. ORF search and gene prediction was performed for the complete plasmid sequence with Lasergene software (DNASTAR, Inc., Madison, WI, USA) and pairwise alignment was analyzed by blastN and blastP homology search using the NCBI database (http://www.ncbi.nlm.nih.gov/blast).

### Nucleotide Sequence Accession Number

The complete sequence of plasmid pNDM-SAL was submitted to GenBank under accession number KP742988.1

## Results

### Identification and Characterization of *bla*_NDM-1_-Positive Isolate

The isolated *Salmonella enterica* was found to be positive for *bla*_NDM-1_, which was confirmed by amplicon sequencing. The sequence of the *bla*_NDM-1_ showed 100% homology with those reported previously (Chen et al., 2011; Ho et al., 2011; Sekizuka et al., 2011; Bonnin et al., 2012; Carattoli et al., 2012; Fu et al., 2012; Dolejska et al., 2013). By serology, this isolate was identified as *S.* Senftenberg presenting antigenic formula as 1,3,19 : g,s,t : -. The isolate was found to be resistant to almost all antibiotics, including nalidixic acid, ciprofloxacin, norfloxacin, ofloxacin, chloramphenicol, streptomycin, azithromycin, sulfamethoxazole/trimethoprim, ampicillin, cefuroxime, ceftriaxone, cefotaxime, ceftazidime, and also to β-lactamase inhibitor combinations (cefotaxime/clavulanate and ceftazidime/clavulanate). However, it was susceptible to tetracycline and showed reduced susceptibility towards imipenem.

### Conjugation and Transfer of Resistance

Plasmid harboring the *bla*_NDM-1_ was transferable to *E. coli* strains J53, XL1-Blue and to other enteric pathogens like *V. cholerae* O1 Ogawa and *S. flexneri* 2a isolated from the diarrheal patients. Conjugation frequencies were observed at higher rates (∼10^-5^ tranconjugants per donor cell) while using *E. coli* J53 and *S. flexneri* 2a as recipients than *E. coli* XL1-Blue (∼10^-6^). The transfer of NDM plasmid into *V. cholerae* O1 Ogawa was even less efficient (∼10^-8^) compared to the other strains (**Table 1**). In addition, the transconjugants acquired additional resistance against β-lactam antibiotics, namely ampicillin, cefuroxime, ceftriaxone, cefotaxime, ceftazidime, and also toward cephalosporin inhibitor combinations like cefotaxime/clavulanate and ceftazidime/clavulanate (**Table 1**). Interestingly, sulfamethoxazole resistance was noticed only in *S. flexneri* transconjugant. Though the CT-*E. coli* J53 was susceptible to sulfamethoxazole/trimethoprim, its MIC was found to increase by 2.7-fold. In CT-*S. flexneri*, the MIC of SXT rose by 31.6-fold, suggesting that sulfamethoxazole resistance was greatly expressed in CT-*S. flexneri* but not in CT-*E. coli* strains.

**Table 1:** Antibiotic susceptibilities of donor, recipients, and transconjugants.

Strain	Bacteria	Resistance profile	MIC value (μg/ml)			
			
			TET	CRO	IPM	SXT
BCH 2406 (Donor)	*Salmonella* Senftenberg	NA, NOR, CIP, OFX, SXT, CHL, AZM, STR, AMP, CXM, CRO, CTX, CTX-CLA, CAZ, CAZ-CLA, IPM (I)	3	>256	6	>32
XL1-Blue (Recipient)	*Escherichia coli*	NA, TET	192	0.5	0.125	
CT-*E. coli* XL1-Blue (Transconjugant)	*E. coli* Transfer frequency^∗^: 3.6 × 10^-6^	NA, TET, AMP, CXM, CRO, CTX, CTX-CLA, CAZ, CAZ-CLA	96	>256	3	
J53 (Recipient)	*E. coli*	AZD		0.5	0.125	0.047
CT-*E. coli* J53 (Transconjugant)	*E. coli* Transfer frequency^∗^: 1.9 × 10^-5^	AMP, CXM, CRO, CTX, CTX-CLA, CAZ, CAZ-CLA, AZD		>256	1.5	0.125
IDH 5313 (Recipient)	*Vibrio cholerae* O1 Ogawa	NA, SXT, STR		0.5	1.5	>32
CT-*V. cholerae* (Transconjugant)	*V. cholerae* O1 Ogawa Transfer frequency^∗^: 1.5 × 10^-8^	NA, SXT, STR, AMP, CXM, CRO, CTX, CTX-CLA, CAZ, CAZ-CLA, IPM		>256	>32	>32
IDH 3077 (Recipient)	*Shigella flexneri* 2a	NA, STR, TET		0.75	0.25	0.38
CT-*S. flexneri* (Transconjugant)	*S. flexneri* 2a Transfer frequency^∗^: 3.7 × 10^-5^	NA, TET, STR, SXT, AMP, CXM, CRO, CTX, CTX-CLA, CAZ, CAZ-CLA, IPM		>256	32	12


NA, nalidixic acid; CIP, ciprofloxacin; NOR, norfloxacin; OFX, ofloxacin; AZM, azithromycin; STR, streptomycin; AMP, ampicillin; CXM, cefuroxime; CRO, ceftriaxone; CTX, cefotaxime; CAZ, ceftazidime; CTX-CLA, cefotaxime/clavulanate; CAZ-CLA, ceftazidime/clavulanate; IPM, Imipenem; SXT, sulfamethoxazole/trimethoprim; CHL, chloramphenicol; TET, tetracycline; AZD, azide; (I) = intermediate resistance.

^∗^Frequency of transfer was calculated as number of transconjugants per donor cell.

The CT-*E. coli* showed reduced susceptibility to carbapenems (**Table 1**). On the contrary, the transconjugants of clinical strains (CT-*V. cholerae* and CT-*S. flexneri*) showed high level resistance to imipenem, far greater than the donor strain *S*. Senftenberg (>5-fold). The MIC values for imipenem were found to be higher in CT-*S. flexneri* (128-fold) than those in CT*-V. cholerae* (>21-fold), CT-*E. coli* XL1-Blue (24-fold), and CT-*E. coli* J53 (12-fold). For ceftriaxone, more than 300-fold rise in MIC was observed for all CTs compared to the respective recipients.

### Plasmid Analysis

**FIGURE 1:**
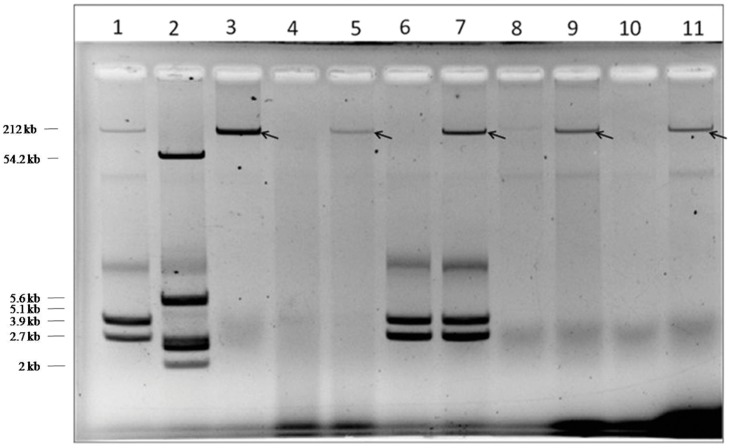
**Plasmid profile of donor, recipient, and transconjugants.** Lanes 1 and 2, *Shigella flexneri* YSH 6000 and *Escherichia coli* K12 V517 were used as reference standards, respectively; Lane 3, BCH 2406: *Salmonella* Senftenberg (donor); Lane 4, IDH 5313: *Vibrio cholerae* O1 Ogawa (recipient); Lane 5, CT-*V. cholerae* (transconjugant); Lane 6, IDH 3077: *S. flexneri* 2a (recipient); Lane 7, CT-*S. flexneri* (transconjugant); Lane 8, XL1-Blue, *E. coli* (recipient); Lane 9, CT-*E. coli* XL1-Blue (transconjugant); Lane 10, *E. coli* J53 (recipient); Lane 11, CT*-E. coli* J53 (transconjugant).

**FIGURE 2:**
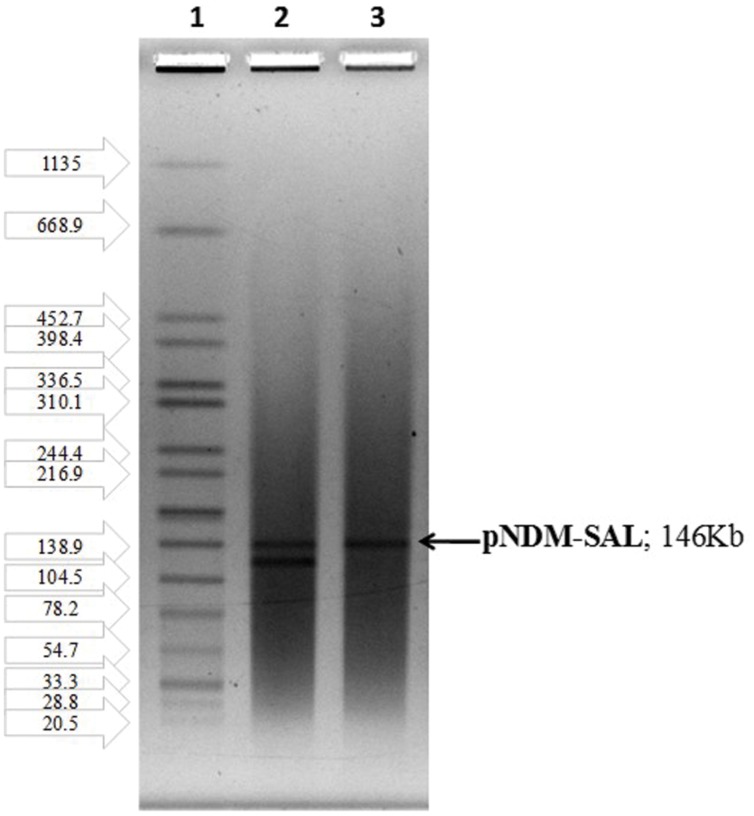
**S1-PFGE indicating the transfer of single plasmid harboring *bla*_NDM-1_: Lane1, H-9812 (PFGE marker strain, *S.* Braenderup H9812); Lane 2, BCH 2406 *S.* Senftenberg (donor); Lane 3, CT-*E. coli* J53 (transconjugant)**.

Plasmid profiles of the donor and transconjugants (**Figure 1**) revealed the transfer of a mega plasmid from *S*. Senftenberg which conferred β-lactam resistance to the recipient strains of *E. coli, V. cholerae*, and *S. flexneri*. NDM-1 PCR assay confirmed the presence of *bla*_NDM-1_ in all the transconjugants. S1-PFGE clearly showed that the resistance phenotype was associated with the transfer of single plasmid of about 146-kb (**Figure 2**) and the NDM-encoding gene was detected by PCR on the plasmid isolated from the transconjugants. Plasmid pNDM-SAL was assigned to the IncA/C incompatibility group using the PCR-based PBRT method.

### Sequence Analysis of Plasmid pNDM-SAL

**FIGURE 3:**
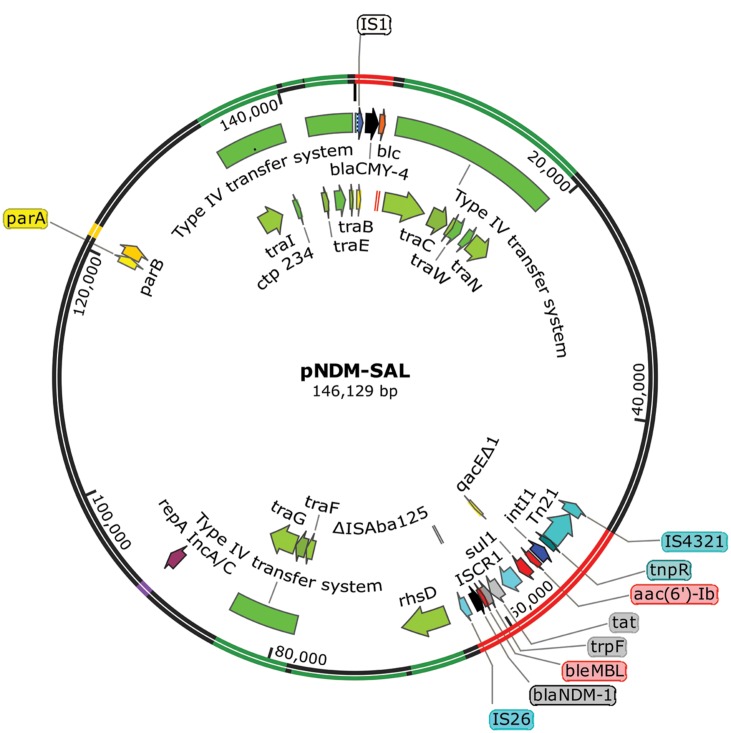
**Salient features of *bla*_NDM-1_ and *bla*_CMY -4_ encoding plasmid pNDM-SAL with transposons and type IV conjugative transfer system.** Starting from the outside, the first circle indicates the coordinate of the complete plasmid circle. Transfer machinery and variable region are showed in green and red, respectively. *rep* and *par* loci are showed in purple and yellow, respectively. The open reading frames (ORFs) were annotated in the inner circle with arrows representing the direction of transcription. β-lactam resistance genes are denoted by black color and other resistance genes as red color. All transposons and IS elements are indicated in shades of blue. ORFs responsible for conjugation are denoted by shades of green.

Whole plasmid showed the plasmid pNDM-SAL to consist of 146.13-kb with an average %GC content of 51.7. In addition to its own replication machinery with partitioning system, it has two separate gene clusters for *bla*_NDM-1_ and *bla*_CMY -4_ (**Figure 3**). It contained 155 predicted coding sequences (CDSs). This plasmid shared extensive homology (99% identity with 98% query coverage) with an IncA/C plasmid, pNDM-1_Dok01 (195.56-kb, described for *E. coli*, accession no. AP012208) including the complete array of genes for replication, type IV conjugative transfer machinery, partition and stabilization, except for the flanking region around *bla*_NDM-1_ and *bla*_CMY -4._ In addition, it had sequence similarity with *Klebsiella* plasmid pNDM-KN (JN157804) and *Citrobacter* plasmid pNDM-CIT (JX182975) showing 99% identity with 87% query coverage and 99% identity with 87% query coverage, respectively.

### Analysis and Comparison of Gene Organization Around *bla*_NDM-1_ and between Other Plasmids

**FIGURE 4:**
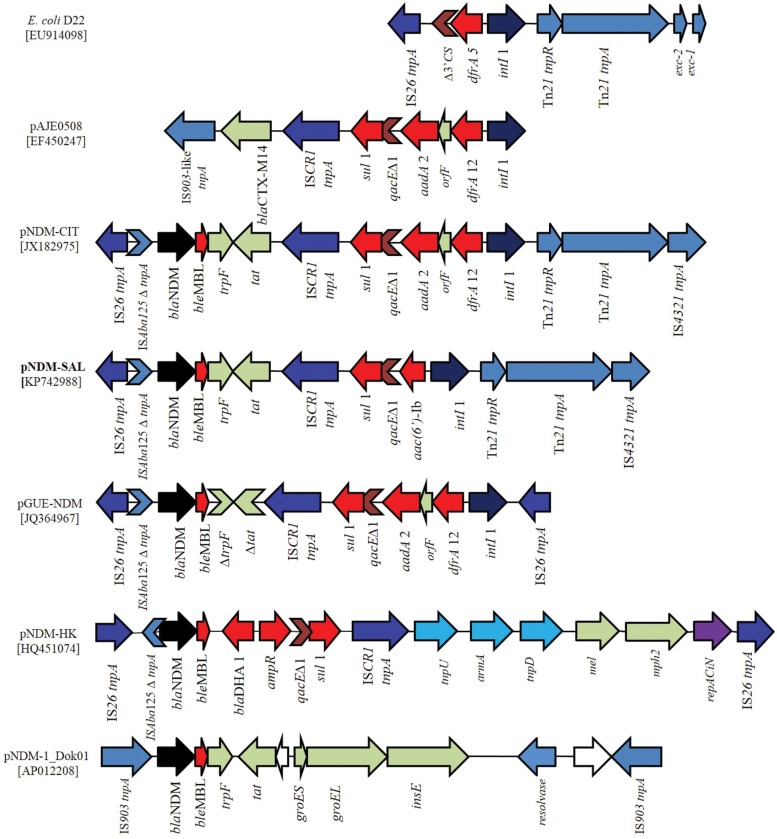
**Schematic representation of the DNA sequences surrounding the *bla*_NDM-1_ genes in pNDM-SAL compared to the sequences in *E. coli* D22 (EU914098), pAJE0508 (EF450247), pNDM-CIT (JX182975), pGUE-NDM (JQ36496), pNDM-HK (HQ451074), and pNDM-1_Dok01 (AP012208).** Arrows showed the direction of transcription. Gaps between the ORFs are indicated by straight line. The same color and label are used to represent homologous genes. *bla*_NDM-1_ are denoted by black color and other resistance genes as red color. All transposons and IS elements are indicated in shades of blue. Other ORFs are denoted by light gray and white. Violet arrow indicates the *repAciN* gene.

The *bla*_NDM-1_ gene was localized in a multidrug resistance region of 15.3-kb (**Figure 4**). This region was bracketed by two different copies of IS elements in inverted orientation, suggesting that the gene was acquired as a composite transposon. Unlike, pNDM-1_Dok01 and pGUE-NDM (87-kb, described in *E. coli*, accession no JQ36496) where *bla*_NDM-1_ gene was flanked by IS*903* and IS*26*, respectively, pNDM-SAL possessed IS*26* and IS*4321* upstream and downstream of the *bla*_NDM-1_. This orientation is much similar to that found in the pNDM-CIT. In between the IS*26* element and the *bla*_NDM-1_ gene, a remnant of IS*Aba125* insertion sequence was identified which comprised –35 promoter sequences leading to the high expression of NDM.

Downstream of the *bla*_NDM-1_, the *ble*_MBL_ gene encoding resistance to bleomycin followed by a truncated phosphorybosilanthranilate isomerase gene (*trpF*), and a twin-arginine translocation pathway signal protein gene (*tat*) was identified. Such an arrangement has been reported previously in other plasmid scaffolds (Sekizuka et al., 2011). Following this, a IS*CR1* element was identified trailed by a class 1 integron containing *aac(6′)-Ib* as its variable resistance gene cassette. Such genetic arrangement has been found in a complex class 1 integron containing IS*CR1* element of ESBL positive *E. coli* (EF450247) and *V. cholerae* (DQ310703). IS*CR1* and IS*26* are known to mediate transposition and/or expression of multiple resistance genes in their close proximity (Bae et al., 2007; Kiiru et al., 2013). Similarly, class 1 integron bearing IS*CR1* was detected in the previously described NDM plasmids, namely pGUE-NDM (JQ364967) and pNDM-CIT (JX182975). The only difference being that the other plasmids harbored *dfrA12-ofrF-aadA2* as their integron resistance gene cassettes (**Figure 4**). However, in all the cases *qacE*Δ1 and *sul*1 gene are present in the integron structure. The *intI*1 gene was followed by the *tnpR* and *tnpA*, the genes of transposon Tn*21* and then by a copy of insertion element IS*4321*, as previously identified in pNDM-CIT (JX182975). Like pNDM-SAL, linkage between Tn*21* transposon bearing class 1 integron and IS*26* element has been seen in NDM negative *E. coli* of human and animal origin (Dawes et al., 2010) and in *E. coli* D22 (EU914098). From the *in silico* analysis, it was not possible to determine the genetic events that led to the formation of this heterogeneous genetic structure. However, it is likely that multiple genetic events contributed to the acquisition of the *bla*_NDM-1_ containing locus by the plasmid.

### The *bla*_CMY_ Module Region

**FIGURE 5:**
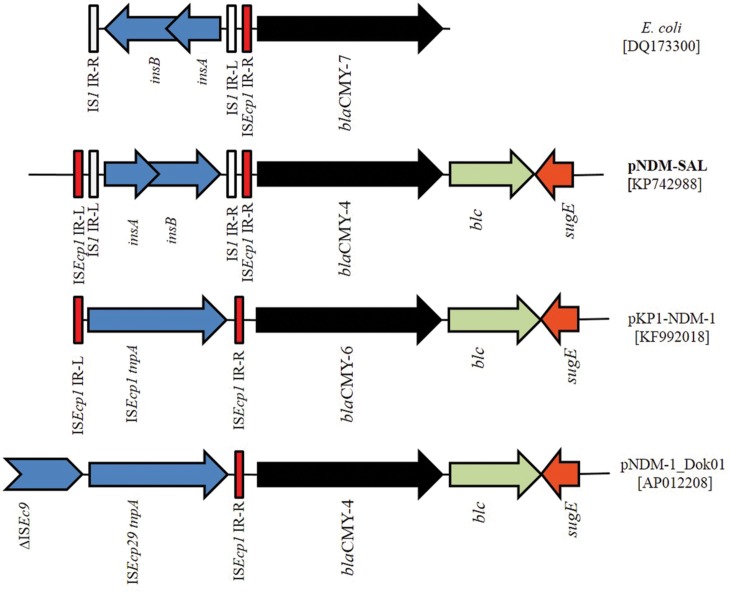
**Schematic representation of the DNA sequences surrounding the *bla*_CMY -4_ genes in pNDM-SAL and comparison with the sequences in *E. coli* (DQ173300), pKP1-NDM-1 (KF992018), and pNDM-1_Dok01 (AP012208).** Arrows indicating *bla*_CMY -4_ (black), *blc* (gray) and *sugE* (orange) are showed according the direction of transcription. Gaps between the ORFs are indicated by straight line. The same color and label are used to represent homologous genes. IS elements are indicated in shades of blue. Red and white color indicates the inverted repeat (IR) of IS*Ecp1* and IS*1*, respectively.

In addition to the *bla*_NDM-1_, pNDM-SAL carried an additional β-lactam resistance gene, *bla*_CMY -4_, distantly located from the NDM harboring composite transposon (**Figure 5**). The *bla*_CMY_ gene was preceded by the IS*1* instead of IS*Ecp1*, as reported previously for other NDM plasmids, pNDM-1_Dok01 and pKP1-NDM-1 (KF992018). Remarkably, *bla*_CMY_ gene associated with IS*1* element was detected before in NDM-negative *E. coli* (DQ173300) with inverted repeats (IRs) of IS*Ecp1* (Hopkins et al., 2006). This makes it tempting to speculate that an intact copy of IS*EcP1* was responsible for the early transposition and mobility of *bla*_CMY -4_ followed by insertion of the IS*1* element; a process similar to what has been reported previously for *E. coli* (DQ173300). Downstream of the *bla*_CMY -4_ gene, *blc* gene encoding outer membrane lipoprotein was detected followed by the *sugE* encoding quaternary ammonium compound resistance protein (**Figure 5**), a feature common in many of the *bla*_CMY_ regions in other NDM-positive plasmids (Kang et al., 2006; Sekizuka et al., 2011). This IS*1*-*bla*_CMY_ module was located within the *tra* locus. Similar insertion with IS*Ecp1* has been demonstrated before (Poole et al., 2009).

## Discussion

The present study revealed EDR features of the pNDM-SAL isolated from *S.* Senftenberg. This finding highlights the ease with which the resistance determinants can move to other enteric pathogens. Firstly, the *bla*_NDM-1_ is located on a broad host range IncA/C plasmid, providing a possible explanation why the pNDM-SAL could be transferred by conjugation to *E*. *coli* (*E. coli* J53 and XL1-Blue) as well as to wild-type strains of *V. cholerae* O1 Ogawa and *S. flexneri* 2a easily. In repeated experiments, CT-*V. cholerae* and CT-*S. flexneri* showed a higher level of imipenem resistance compared to the donor strains. This could be hypothesized to be due to “possibly” a higher copy number of the plasmid carrying the gene in the recipient than in the donor strains. In other words due to “gene dosage” effect. There could be other possible explanations as well. Examples of such phenomenon, transconjugants showing higher drug resistance are documented in the literature (Ouellette et al., 1988; Petroni et al., 2002).

Since IncA/C plasmids possess highly mobile nature, it has commonly been found in NDM harboring bacteria, especially among the members of Enterobacteriaceae (Ghosh et al., 2014). The size of plasmid varies considerably, ranging from 35.9 to 400-kb (Ghosh et al., 2014) with certain shared genetic background. A recent study has shown that the NDM carrying plasmids from Enterobacteriaceae at New Delhi, shared the replicon type IncA/C with140-kb in size (Walsh et al., 2011). Large plasmids belonging to the IncA/C group have received increased attention, primarily due to their ability to confer resistance to a diverse group of antimicrobial agents (Fernandez-Alarcon et al., 2011). Transmission of pNDM-SAL was also found to be closely associated with transfer of multi drug resistance. Since clinical strains of *Vibrio* or *Shigella* are typically resistant to some antimicrobials, transfer of pNDM-SAL carrying *bla*_NDM-1_ and other genes could make them multi resistance. This situation is of major concern in the clinical management of infections. Since the pNDM-SAL harbored antibiotic resistance genes are clustered in a few integration hotspots within complex genetic structures, they may be able to acquire novel antibiotic resistance genes through homologous recombination (Doublet et al., 2012). In the pNDM-SAL, *rhs* locus remains near *bla*_NDM-1_ with a phage-integrase and hence they are likely to be the hotspots for integration of accessory genes within the IncA/C plasmids (Carattoli et al., 2012).

In pNDM-SAL, *bla*_NDM-1_ was located between IS*26* and IS*4321*. Similar genetic arrangements have previously been observed in other *bla*_NDM-1_ bearing plasmids (Sekizuka et al., 2011; Bonnin et al., 2012; Dolejska et al., 2013). Comparison of the flanking regions of *bla*_NDM-1_ present in different plasmids suggests that different genetic events may have supported acquisition of this gene in different plasmids (Ghosh et al., 2014). In pNDM-SAL, the linkage of *bla*_NDM-1_ with IS*26* creates a condition favorable for the mobilization of *bla*_NDM-1_. Interestingly, in many NDM negative *E. coli* strains, arrangement of class 1 integron between Tn*21* transposon and IS*26* was a characteristic feature (Dawes et al., 2010) as noticed in the pNDM-SAL. The IS*26* element, a member of the IS*6* family, is widespread among Enterobacteriaceae and exists adjacent to the β-lactamases region, which is a part of transposon-like structure in many plasmids (Yu et al., 2006; Ho et al., 2011). Similar to the other plasmids such as *E. coli* DVR22 (JF922606), pNDM-HK (HQ451074), pKpANDM-1 (FN396877) and p271A (JF785549), a truncated IS*Aba125* has been identified upstream of *bla*_NDM-1_ in pNDM-SAL. Interestingly, in *Acinetobacter baumanii* isolates of African origin, the NDM encoding gene was found within the Tn*125*- like or IS*Aba125* (Bonnin et al., 2013). This suggests that the IS*Aba125* insertion sequence has possibly played an important role in the mobilization of *bla*_NDM-1_ from a common progenitor and that event was followed by subsequent transfer events mediated by the other insertion elements like IS*26* as seen in *Acinetobacter* spp. and Enterobacteriaceae (Ghosh et al., 2014). In pGUE-NDM (*E. coli*, JQ364967) and pNDM-MAR (*Klebseilla*, JN420336) NDM regions are fully bracketed by IS*26.* But pNDM-SAL possesses IS*4321* and Tn*21* tranposases in one end similar to what is found in the pNDM-CIT (*Citrobacter*, JX182975). Instead of *bla*_DHA-1_ gene commonly found downstream of many NDM plasmids, eg. pNDM-HK (*E. coli*, HQ451074) the pNDM-SAL IS*CR1* bearing class 1 integron has been inversely located (**Figure 4**). The association between *ble*_MBL_ and *bla*_NDM_ appears to be strong as they are expressed by a common promoter. This may be the reason why both the genes transfer *en bloc* (Fu et al., 2012; Ghosh et al., 2014).

Based on the genetic environment of *bla*_NDM-1_ in pNDM-SAL, we hypothesize that (i) a common transposon structure with IS*Aba125* could be responsible for the early acquisition of *bla*_NDM-1,_ (ii) by extensive genetic rearrangement; it was captured in a Tn*21* linked complex class 1 integron bearing IS*CR1*, (iii) later on it was bracketed by two IS elements, namely IS*4321* preceding the Tn*21* transposase and IS*26* truncating the IS*Aba125*.

The plasmid pNDM-SAL has similarity to other NDM-plasmids, which harbor *bla*_CMY -4_ and the complex class 1 integron carrying several antibiotic resistance-conferring genes (Toleman et al., 2006a,b). AmpC-like cephalosporinase (*bla*_CMY_) genes that have been frequently mobilized by IncA/C-type plasmids, could be identified in *E. coli* and *Salmonella* isolates not only of human but also of animal origins in the United States, Canada, and Europe (Winokur et al., 2001; Carattoli et al., 2012). The evolutionary relationship between NDM-1-bearing plasmids and the IncA/C plasmids carrying *bla*_CMY_ suggests that IncA/C *bla*_CMY_-carrying plasmid could have acquired the *bla*_NDM-1_ within its scaffold due to a secondary event (Carattoli et al., 2012).

From the data presented in this paper it can be seen that plasmid pNDM-SAL possesses many interesting features as it contain gene encoding the metallo-β-lactamase NDM-1, which is positively associated with multidrug resistance (**Figure 3**). Besides, pNDM-SAL harbors a large arsenal of genetic elements (integrons, transposons, and IS*CR*s), giving it the ability to acquire and disseminate antibiotic resistance genes. However, the extent of carbapenem resistance due to the presence of NDM plasmid varied in different hosts. The trend of antimicrobial susceptibility is shifting toward old generation antibiotics, which are less commonly used in recent years (Meziane-Cherif and Courvalin, 2014). In many studies, it was shown that NDM producers were susceptible to old generation antibiotics such as chloramphenicol, tetracycline etc. (Abdul Rahim et al., 2015; Uppu et al., 2015). In our finding, excerpt for tetracycline, the NDM-positive *S.* Senftenberg were resistant to most of the old generation antibiotics such as ampicillin, trimethoprim, sulfamethoxazole, streptomycin, nalidixic acid, and chloramphenicol. It appears that the maintenance of resistance to any given antibiotic may vary from species to species.

From our data it can be inferred that the strain *S.* Senftenberg probably was not the natural host for this NDM plasmid but once acquired, the plasmid it had the ability to transfer it to a broad range of pathogenic and non-pathogenic Gram-negative bacteria, an observation which merits serious and important attention to our findings.

## Author Contributions

AS, GP, and GC isolated and identified the pathogens, performed phenotypic and genetic analysis. AG and TR analyzed the data, conceived the idea, and wrote the manuscript. All authors were involved in the compilation of the report and approved the final version.

## Conflict of Interest Statement

The authors declare that the research was conducted in the absence of any commercial or financial relationships that could be construed as a potential conflict of interest.
